# Supplementary material for: Repeated semen exposure decreases cervicovaginal SIVmac251 infection in rhesus macaques
Source: Nat Commun. 2019 Aug 21;10:3753. doi: 10.1038/s41467-019-11814-5 (PMC6704120; doi:10.1038/s41467-019-11814-5)
Supplement: Supplementary file 1 — Supplementary Information [file 41467_2019_11814_MOESM1_ESM.pdf]

Repeated Semen Exposure Decreases Cervico-Vaginal SIVmac251 infection in Rhesus Macaques

Supplementary Information

Abdulhaqq, et al.

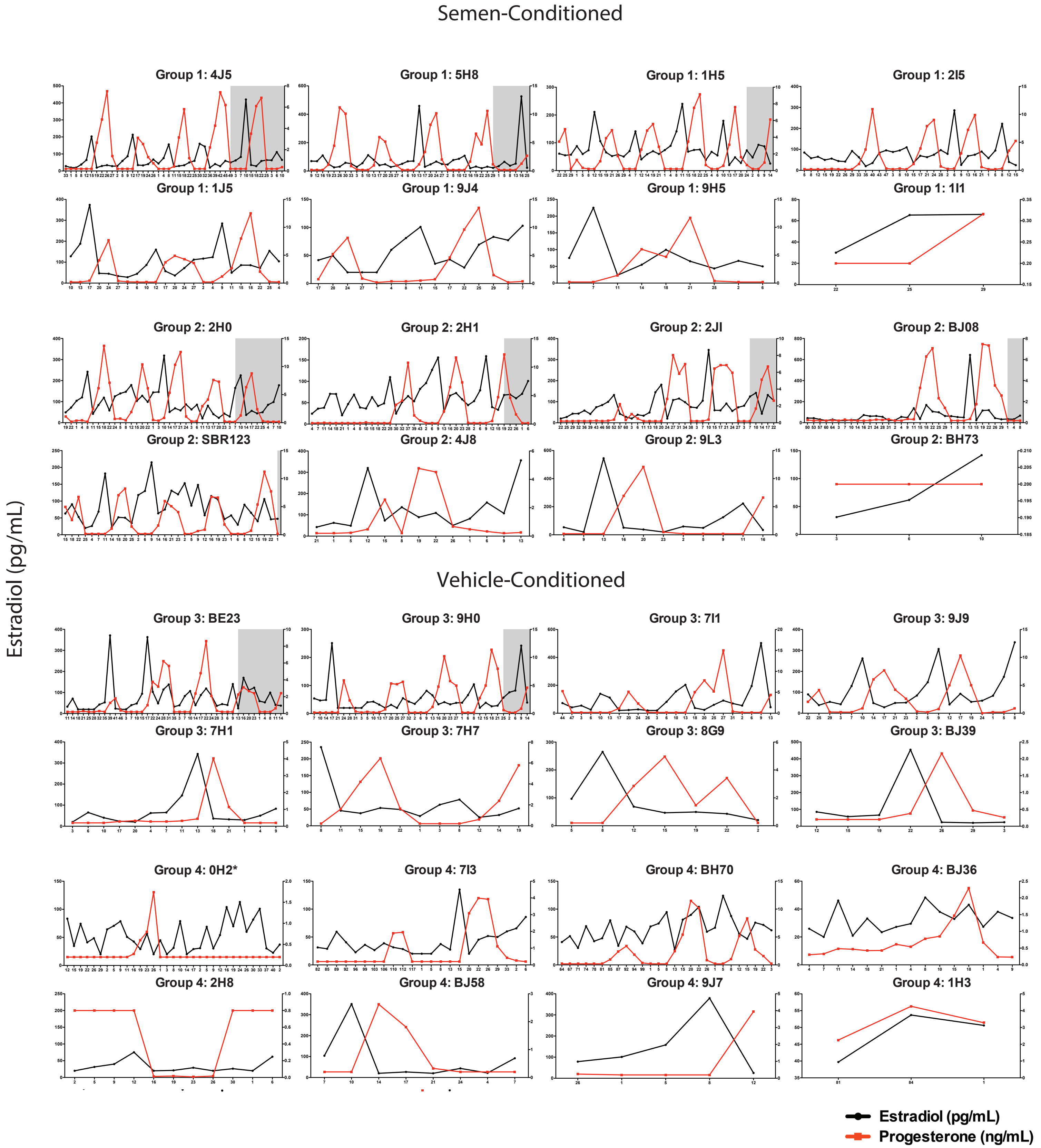

Supplementary Figure 1: **Estradiol and progesterone hormone cycling throughout the low-dose chal-lenge period.** All animals cycled throughout the study with cycle periods between 24-45 days. At the start of the study, several animals had still not started normal summer cycling (7I3, BH70, 2JI, BJ08). These animals were not predisposed to early infection and resumed normal cycling before infection. One animal 0H2 never established normal cyling and also remained uninfected during both the low-dose and high-dose challenge periods. Estradiol graphed to left axis and progesterone to right axis. Graphs begin at start of challenge and end at detected positive viral. Grey indicates the end of the low-dose challenge period. Menstrual period was deter-mined to end at first appearance of menses. Primary data in Data Source File.

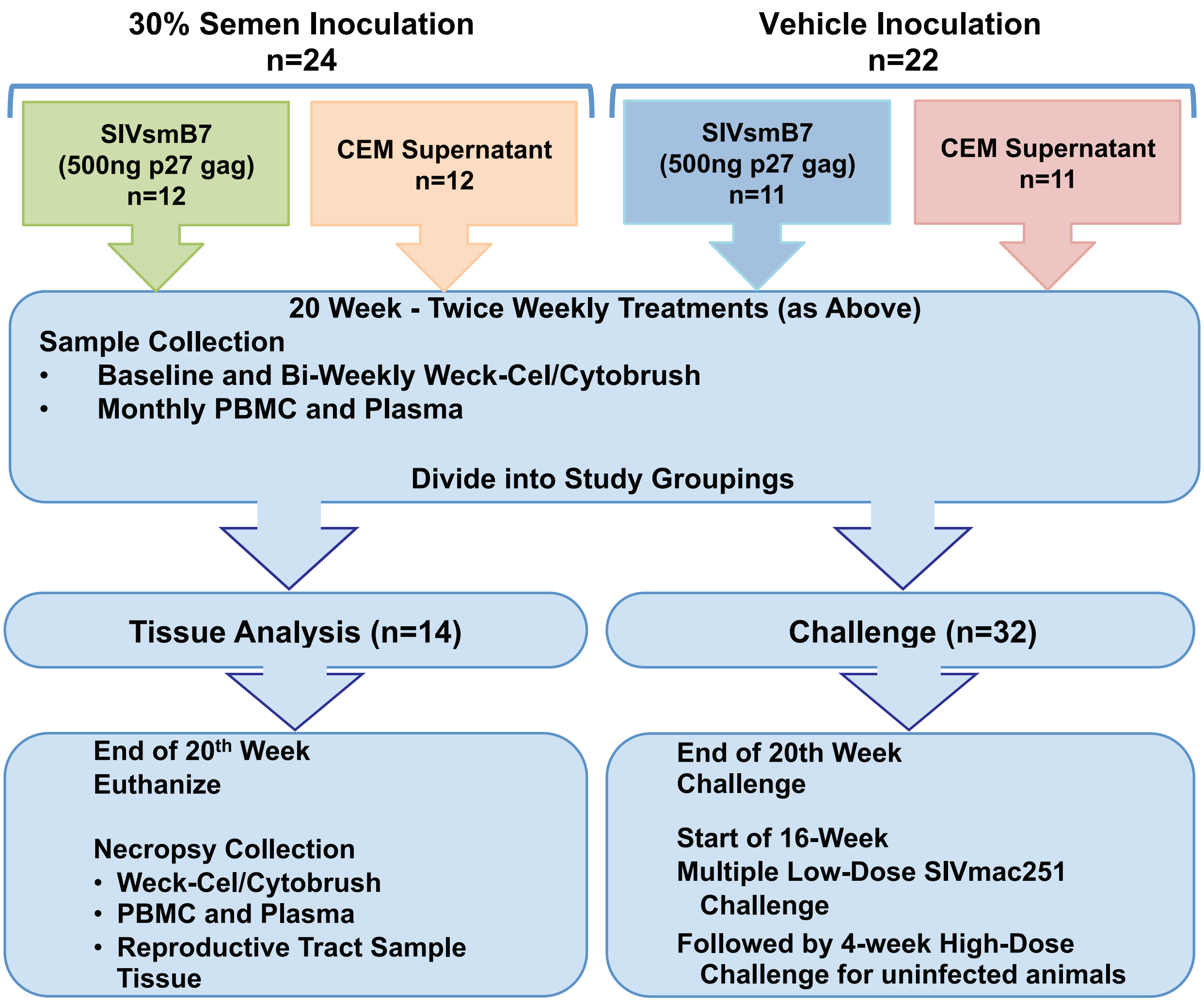

Supplementary Figure 2: Experimental Overview.

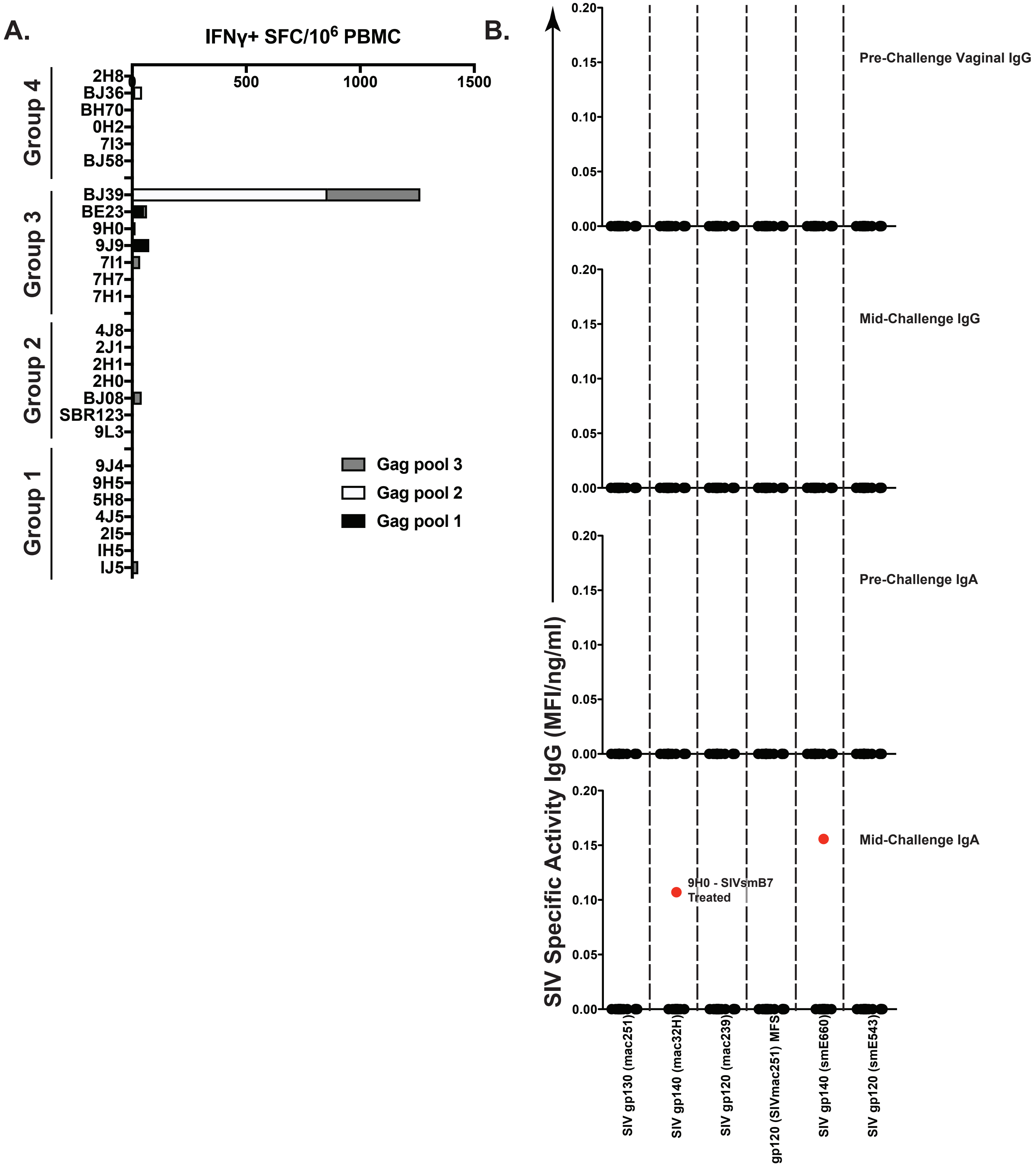

Supplementary Figure 3: Assessment of SIV-specific Immune Responses. (A) 3 pools were made consisting of 15-mers overlapping by 11 that stretched the SIVmac32H gag protein. These pools were used in an ELISpot assay to test for peripheral blood SIV-specific T-cells for 28 of 32 animals 2 weeks prior to each individual animals' SIV infection. Of these only one animal in group 3, treated with SIVsmB7 and vehicle, had detectable gag responses. (B) Binding Antibody Multiplex Assay (BAMA) was done to detect SIV-specific antibody after conditioning and at mid-challenge for all 32 animals. At no point during the study did any animal have detectable SIV-specific IgG despite prolonged exposure to SIVsmB7. Although no animal developed SIV-specific IgA by the 20-week time point, by week 30 animal 9H0 had developed a very low titer of SIV-specific IgA that was cross-re-active to mac32H and smE660. Primary data can be found in the Data Source File.

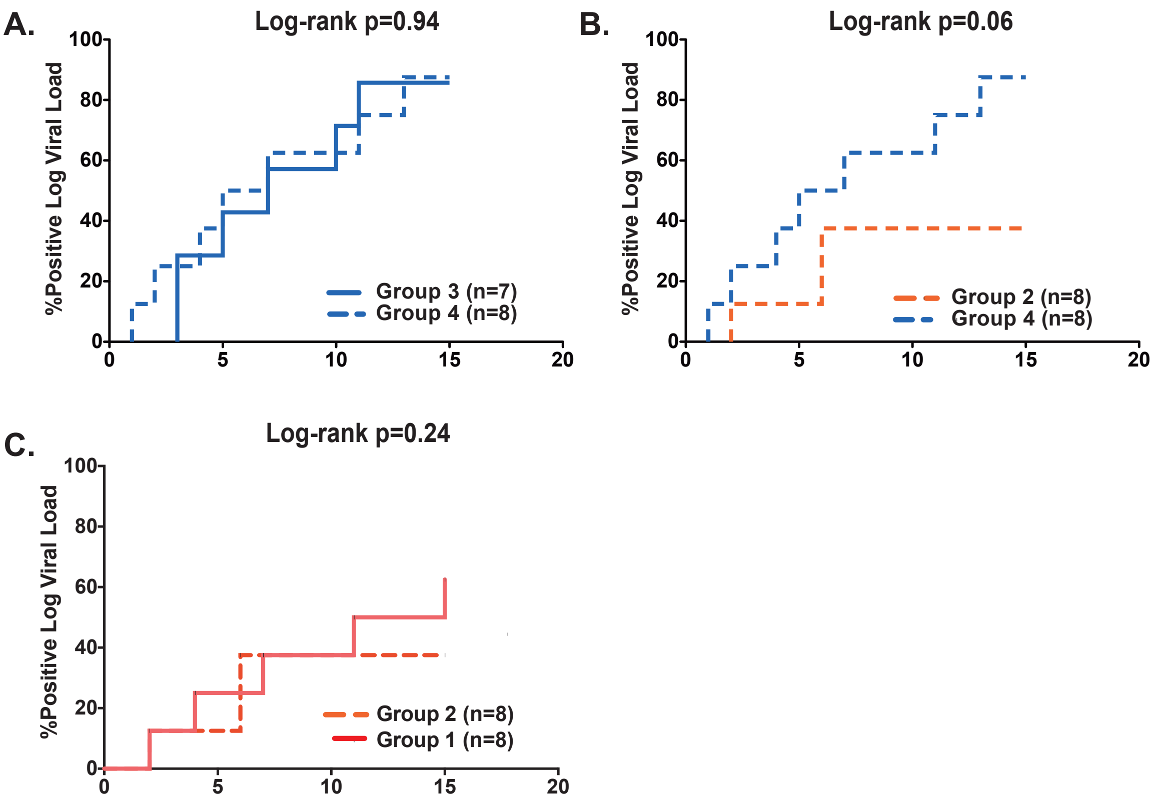

Supplementary Figure 4: Semen not SIVsmB7 administration lowers suscetibility to SIVmac251. (**A**) Direct comparison of animals receiving SIVsmE660 derivative SIVsmB7 as compared to those receiving CEM_{sup} (log-rank p=0.94). (**B**) Comparison of semen-receiving animals to animals receiving CEM_{sup} alone (p=0.06) (**C**) Comparison of semen/CEM_{sup} receiving animals to animals receiving semen/SIVsmB7 (p=0.24). Primary data in Data Source File.

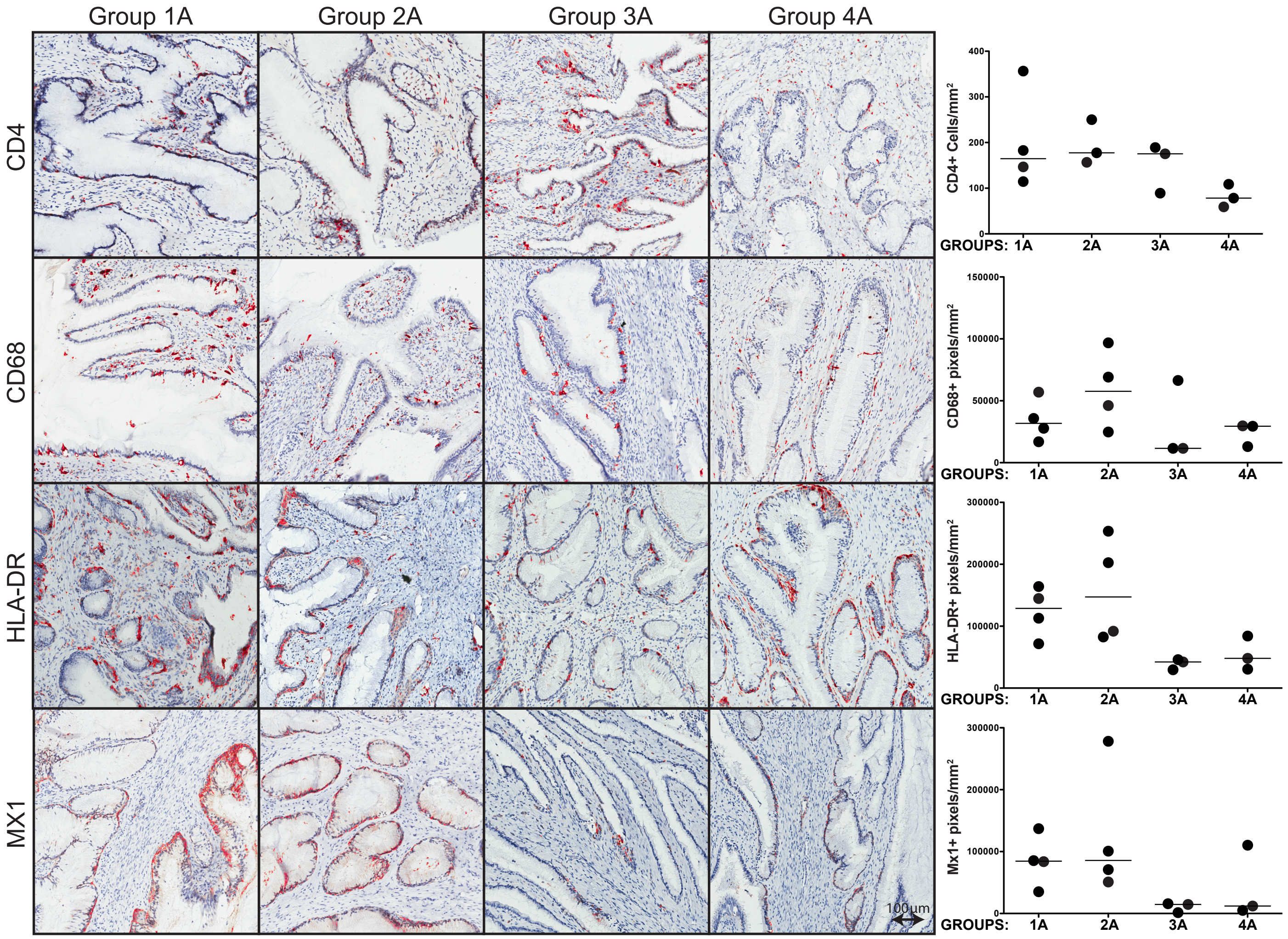

Supplementary Figure 5: Immune infiltrates and activation within endocervical tissue after semen and/or SIVsmB7 conditioning. Amongst markers assessed, SIVsmB7 may have had some impact on CD4+ infiltration; however, due to the size of the effect and the relatively small sample size, statistical confirmation could not be reached. Otherwise, SIVsmB7 had no noticeable impact on immune infiltrates or activation markers. However, HLA-DR and Ki67 appeared to be segregated based on semen exposure. Group 1A: n=4. Group 2A: n=4 (except for CD4 n=3). Group 3A: n=3. Group 4A: n=3. Brown IHC staining was pseudo-colored red for clarity as done in Figure 2. Primary data in Data Source File.

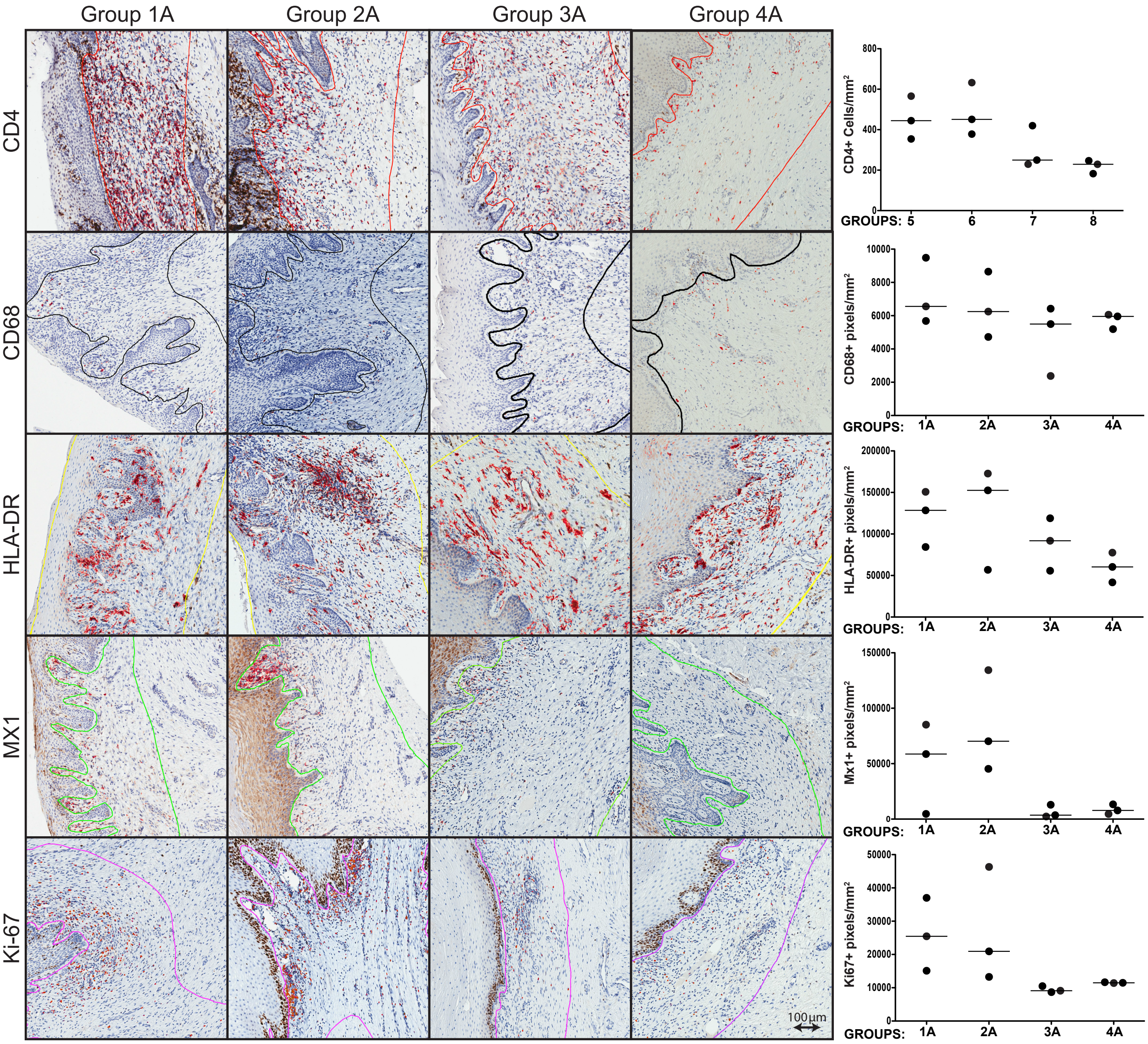

-

Supplementary Figure 6: Immune infiltrates and activation within ectocervical tissue after Semen and/or SIVsmB7 conditioning. Amongst markers assessed, SIVsmB7 appeared to have no significant impact on marker distribution. However, CD4, Mx1, and Ki67 appeared to be segregated based on semen exposure. Group 1A: n=3. Group 2A: n=3. Group 3A: n=3. Group 4A: n=3. The quantified area for each tissue is outlined. Brown IHC staining was pseudo-colored red for clarity as done in Figure 2.

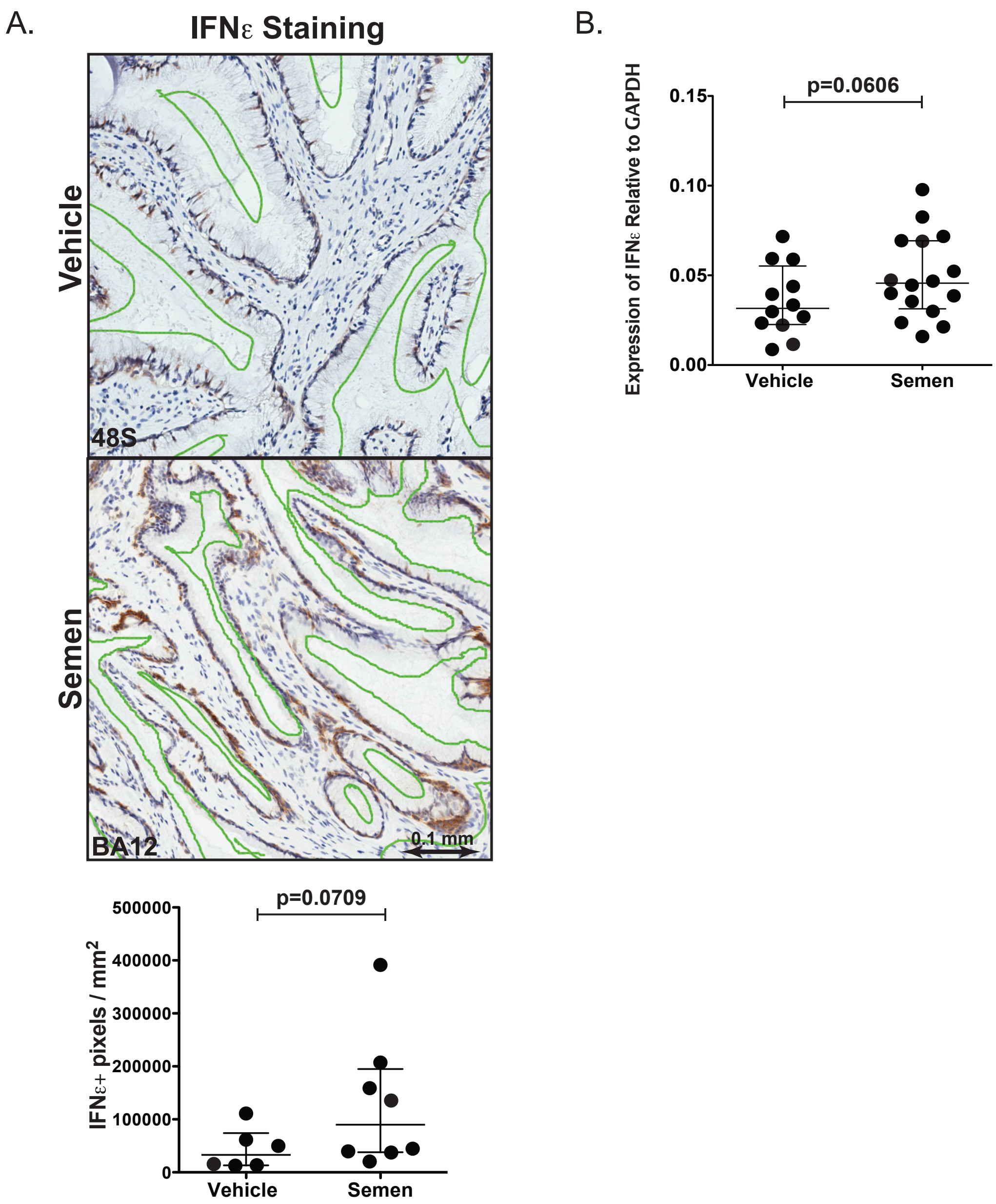

Supplementary Figure 7: Interferon epsilon expression in the FRT of semen or vehicle conditioned animals. (A) Immuno-histochemical staining of endocervical tissue highlighted a trend of increased expres-sion (Mann-Whitney p=0.0709) in semen-treated animals as compared to vehicle (bottom). IFNε staining was confined to the columnar epithelium (semen n=8 / vehicle n=6). **(B)** qPCR of vaginal and cervical tissues from each of the animals showed a similar trend of increased IFNε expression (Mann-Whitney p=0.06). Pairwise analysis was done by one-tailed Mann-Whitney tests. Primary data in Data Source File.

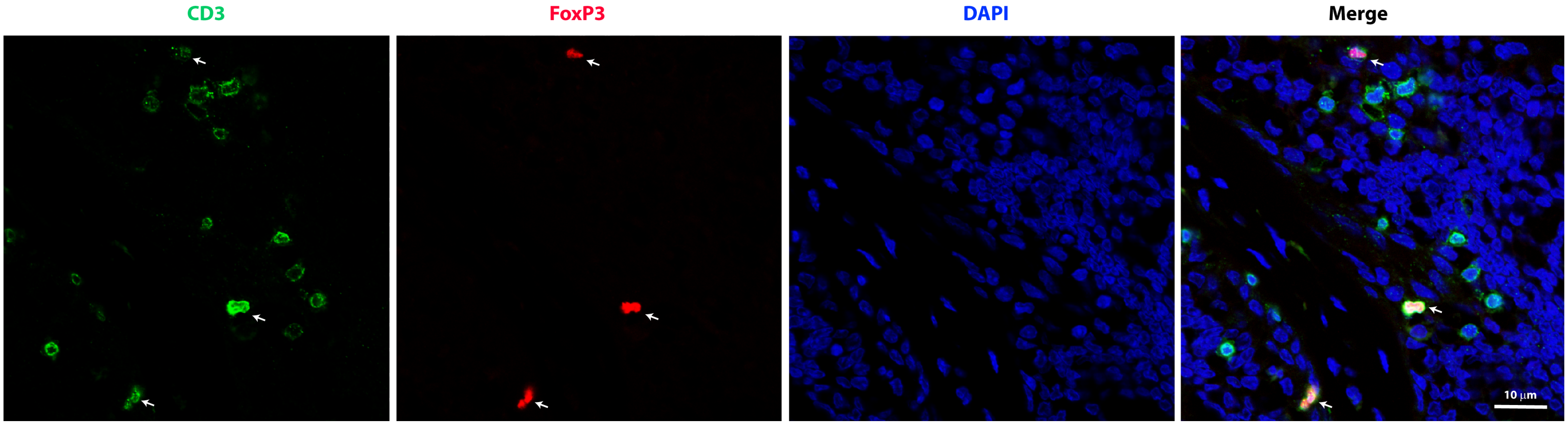

Supplementary Figure 8: FoxP3 staining of the lamina propria of FRT tissues is restricted to CD3+ T-cells. Ectocervical tissue from semen-treated animal (Group 5: BA12) was stained for CD3-FITC (Panel 1), FoxP3-Cy3 (Panel 2), and DAPI (Panel 3). The merge of all stainings is shown in Panel 4.

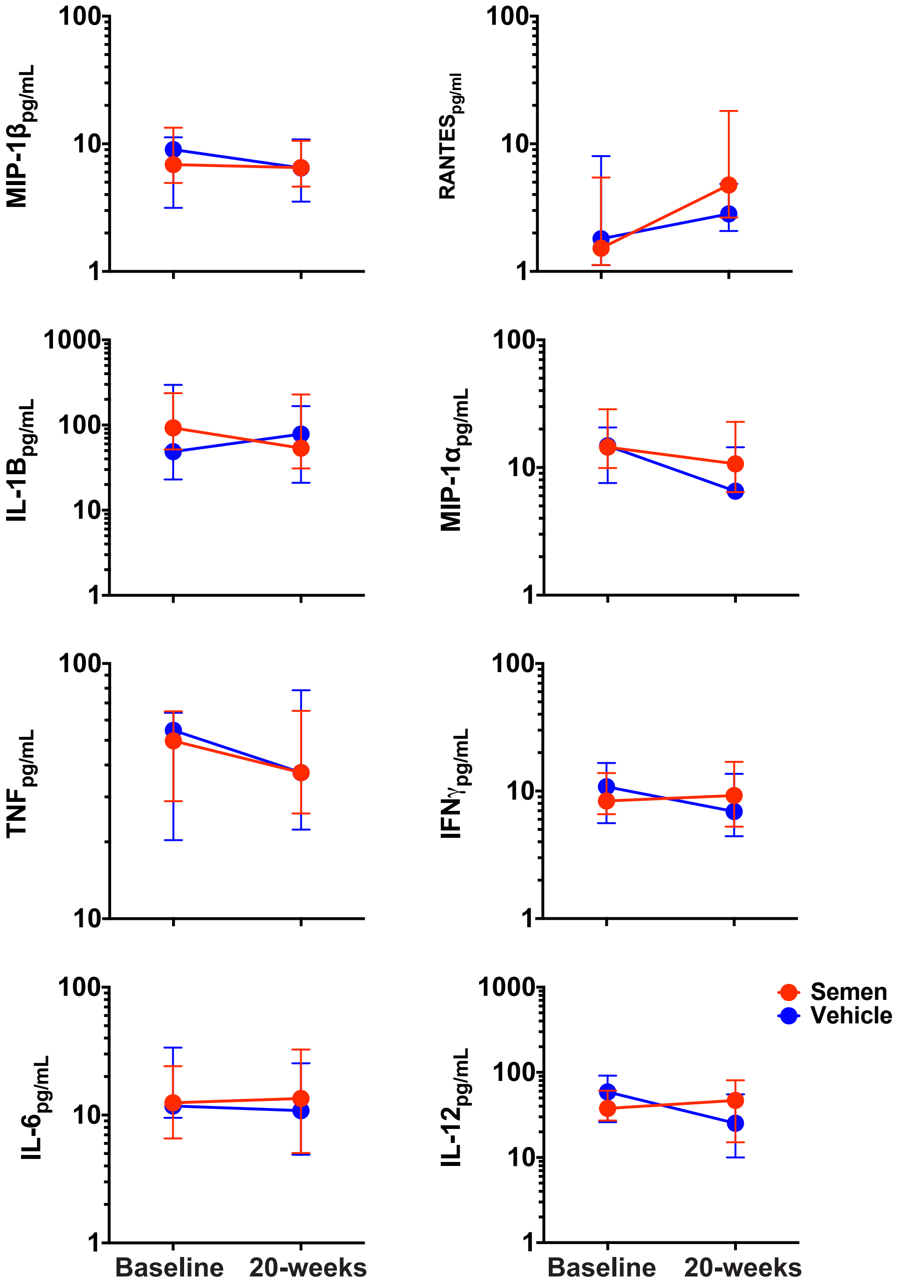

Supplementary Figure 9: Despite alterations in immune cell infiltration intravaginal administration of human semen does not alter intravaginal cytokine/chemokine levels. Cervico-vaginal fluid collected before animal manipulations and after 20-week intravaginal semen inoculation was used in a luminex assay to assess changes in inflammatory cytokine and chemokine secretion in response to inoculations. Levels for IL-1B, IL-6, IL-12, TNF, IFNdid not significantly change between baseline and 20-week timepoints for semen-conditioned animals and levels were not significantly different from vehicletreated animals. (Semen n=16; Vehicle n=15) Primary data in Data Source File.

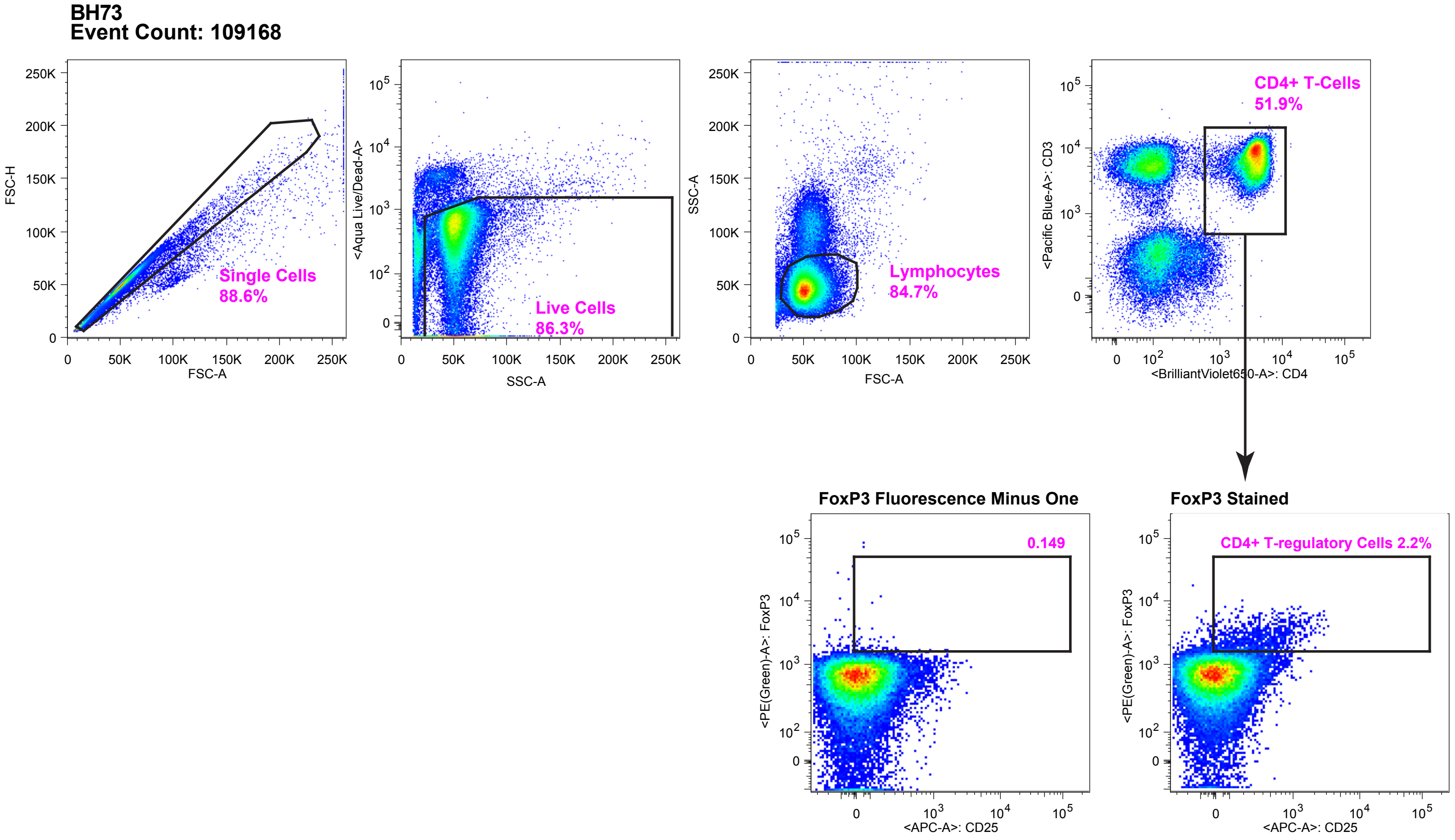

Supplementary Figure 10: Example CD4+ T-regulatory Gating. Cells were gated as indicated. CD4+ T-regu-latory cells were defined as CD3+, CD4+, CD25+, FoxP3+ cells. FoxP3 gating was based off a Fluorescence Minus One (FMO) staining control. These data were used for Supplementary Table 3.

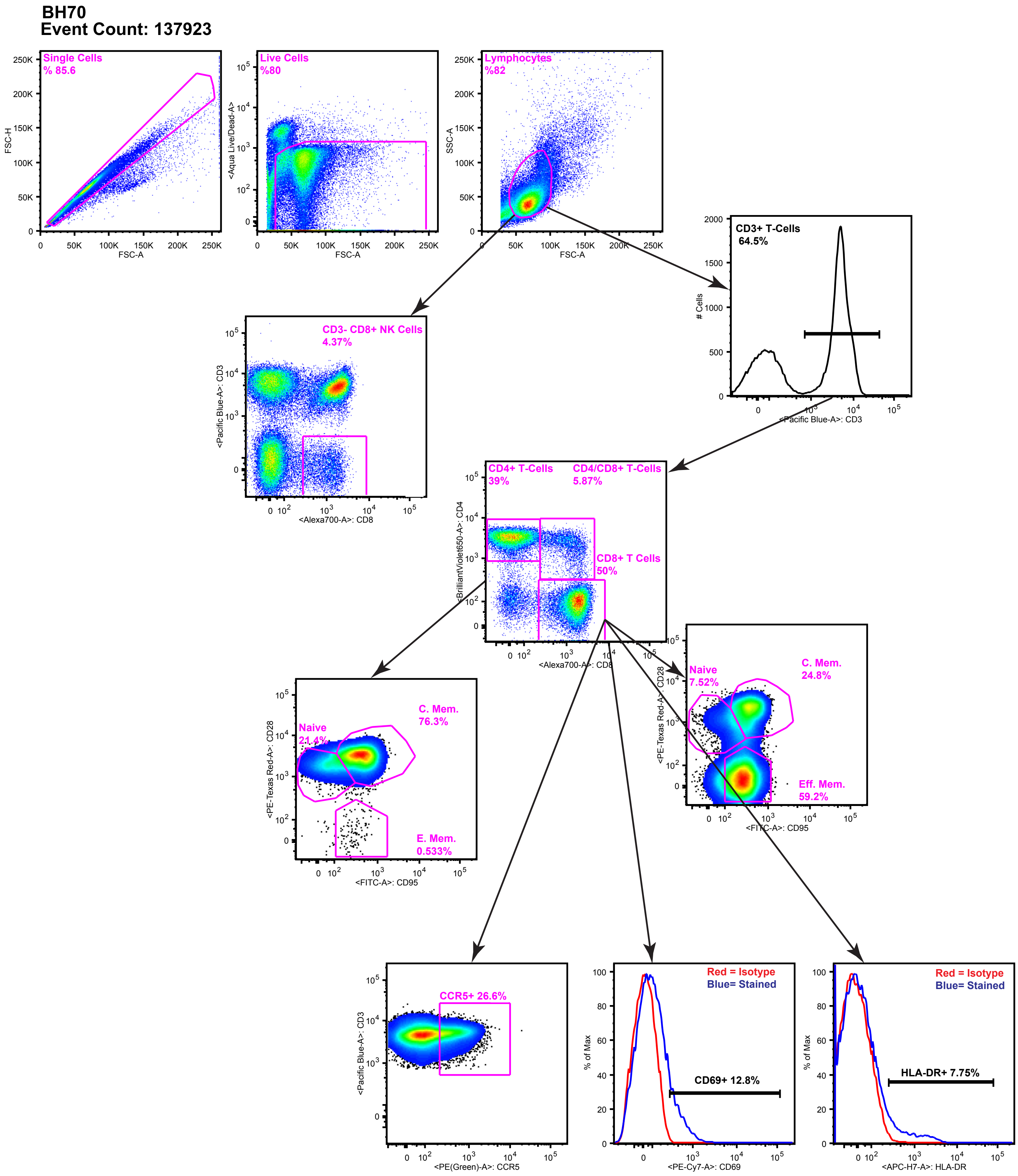

Supplementary Figure 11: Example Gating for T & NK Cell Subsets. Cells were gated as indicated. Gating for memory subsets was based off of unstained controls. Gating for activation markers is exampled with CD8+cells (the same gating strategy was used for CD4). Gating for HLA-DR, CD69, CCR5, and CD25 [not pictured] were all based off of a combination of isotype and FMO controls. CCR5 data were used for Figure 2B, all other data was used for analysis seen in Supplementary Table 3.

9H0
Event Count: 183540

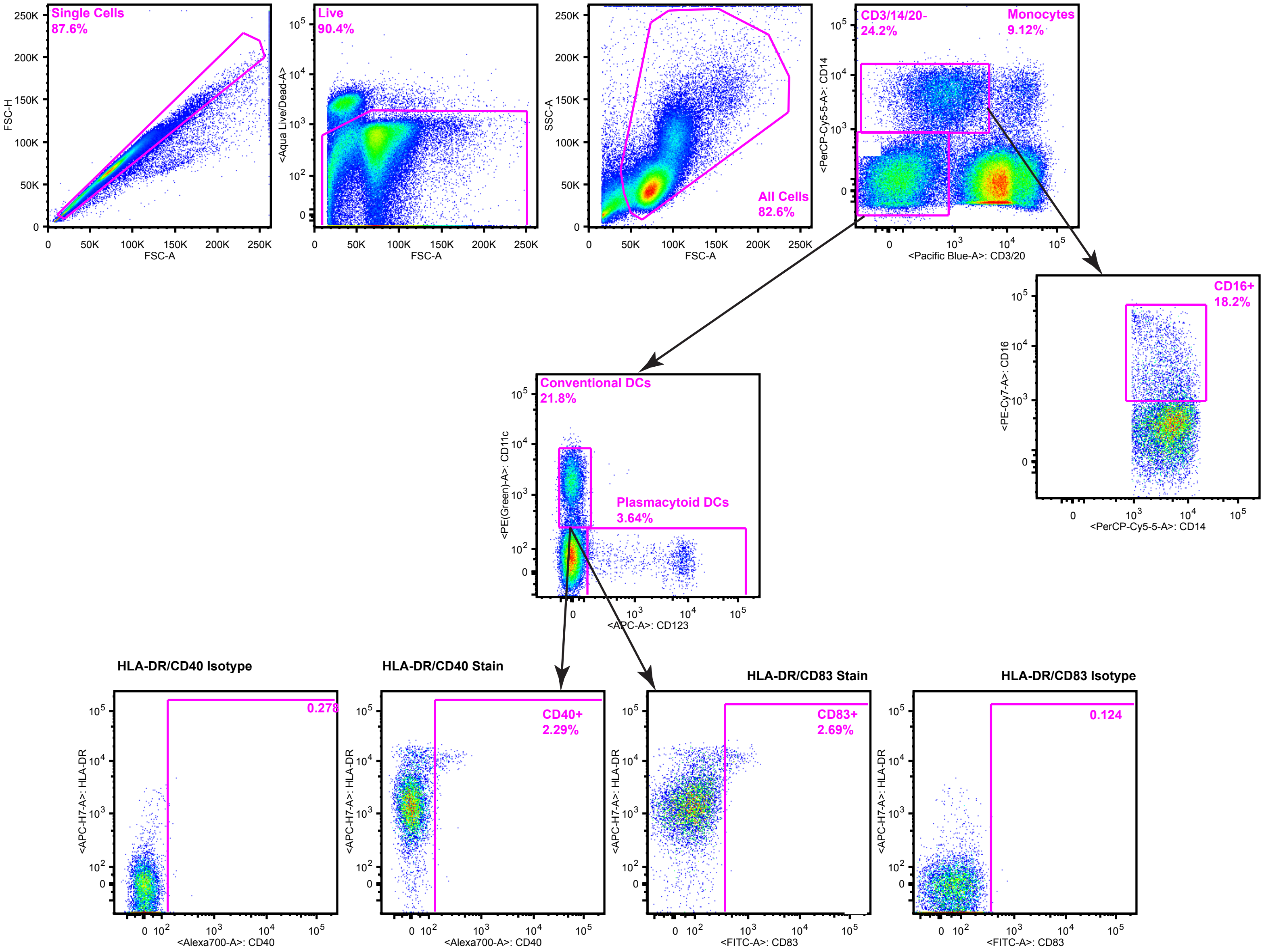

Supplementary Figure 12: Example Gating for Monocytes and DC Subsets Subsets. Cells were gated as indicated. Gating for activation/maturation markers is exampled with conventional dendritic cells (cDC)[the same gating was done for pDC].Gating for HLA-DR, CD40, and CD83 were all based off of isotype controls. These data were used for Supplementary Table 3.

Supplementary Table 1: Detailed Challenge Schedule

Week(s)	Biweekly Treatment*	Inoculum	Group 1: Semen/SIVsmB7	Group 2: Semen/CEM	Group 3: Control/SIVsmB7	Group 4: Control/CEM
1-20	Monday-Tuesday/Thursday-Friday	Dilutant	Semen 150µL	Semen 150µL	RPMI 1640 150µL	RPMI 1640 150µL
		Virus or Mock	500 ng p27 SIVsmB7	CEMx174 Supernatant	500 ng p27 SIVsmB7	CEMx174 Supernatant
**21-22**	Monday-Tuesday	**Dilutant**	Semen 150µL	Semen 150µL	RPMI 1640 150µL	RPMI 1640 150µL
		**Challenge Virus**	200 $TCID_{50}$ SIVmac251	200 $TCID_{50}$ SIVmac251	200 $TCID_{50}$ SIVmac251	200 $TCID_{50}$ SIVmac251
	Thursday-Friday	**Dilutant**	Semen 150µL	Semen 150µL	RPMI 1640 150µL	RPMI 1640 150µL
		**Virus or Mock**	500 ng p27 SIVsmB7	CEMx174 Supernatant	500 ng p27 SIVsmB7	CEMx174 Supernatant
**23 Rest Week**	Monday-Tuesday/Thursday-Friday	**Dilutant**	Semen 150µL	Semen 150µL	RPMI 1640 150µL	RPMI 1640 150µL
		**Virus or Mock**	500 ng p27 SIVsmB7	CEMx174 Supernatant	500 ng p27 SIVsmB7	CEMx174 Supernatant
**24-25**	Monday-Tuesday	**Dilutant**	Semen 150µL	Semen 150µL	RPMI 1640 150µL	RPMI 1640 150µL
		**Challenge Virus**	400 $TCID_{50}$ SIVmac251	400 $TCID_{50}$ SIVmac251	400 $TCID_{50}$ SIVmac251	400 $TCID_{50}$ SIVmac251
	Thursday-Friday	**Dilutant**	Semen 150µL	Semen 150µL	RPMI 1640 150µL	RPMI 1640 150µL
		**Virus or Mock**	500 ng p27 SIVsmB7	CEMx174 Supernatant	500 ng p27 SIVsmB7	CEMx174 Supernatant
**26 Rest Week**	Monday-Tuesday/Thursday-Friday	**Dilutant**	Semen 150µL	Semen 150µL	RPMI 1640 150µL	RPMI 1640 150µL
		**Virus or Mock**	500 ng p27 SIVsmB7	CEMx174 Supernatant	500 ng p27 SIVsmB7	CEMx174 Supernatant
**27-28**	Monday-Tuesday	**Dilutant**	Semen 150µL	Semen 150µL	RPMI 1640 150µL	RPMI 1640 150µL
		**Challenge Virus**	400 $TCID_{50}$ SIVmac251	400 $TCID_{50}$ SIVmac251	400 $TCID_{50}$ SIVmac251	400 $TCID_{50}$ SIVmac251
	Thursday-Friday	**Dilutant**	Semen 150µL	Semen 150µL	RPMI 1640 150µL	RPMI 1640 150µL
		**Virus or Mock**	500 ng p27 SIVsmB7	CEMx174 Supernatant	500 ng p27 SIVsmB7	CEMx174 Supernatant
**29 Rest Week**	Monday-Tuesday/Thursday-Friday	**Dilutant**	Semen 150µL	Semen 150µL	RPMI 1640 150µL	RPMI 1640 150µL
		**Virus or Mock**	500 ng p27 SIVsmB7	CEMx174 Supernatant	500 ng p27 SIVsmB7	CEMx174 Supernatant
**30-31**	Monday-Tuesday	**Dilutant**	Semen 150µL	Semen 150µL	RPMI 1640 150µL	RPMI 1640 150µL
		**Challenge Virus**	400 $TCID_{50}$ SIVmac251	400 $TCID_{50}$ SIVmac251	400 $TCID_{50}$ SIVmac251	400 $TCID_{50}$ SIVmac251
	Thursday-Friday	**Dilutant**	Semen 150µL	Semen 150µL	RPMI 1640 150µL	RPMI 1640 150µL
		**Virus or Mock**	500 ng p27 SIVsmB7	CEMx174 Supernatant	500 ng p27 SIVsmB7	CEMx174 Supernatant
**32 Rest Week**	Monday-Tuesday/Thursday-Friday	**Dilutant**	Semen 150µL	Semen 150µL	RPMI 1640 150µL	RPMI 1640 150µL
		**Virus or Mock**	500 ng p27 SIVsmB7	CEMx174 Supernatant	500 ng p27 SIVsmB7	CEMx174 Supernatant
**33-34**	Monday-Tuesday	**Dilutant**	Semen 150µL	Semen 150µL	RPMI 1640 150µL	RPMI 1640 150µL
		**Challenge Virus**	400 $TCID_{50}$ SIVmac251	400 $TCID_{50}$ SIVmac251	400 $TCID_{50}$ SIVmac251	400 $TCID_{50}$ SIVmac251
	Thursday-Friday	**Dilutant**	Semen 150µL	Semen 150µL	RPMI 1640 150µL	RPMI 1640 150µL
		**Virus or Mock**	500 ng p27 SIVsmB7	CEMx174 Supernatant	500 ng p27 SIVsmB7	CEMx174 Supernatant
**35 Rest Week**	Monday-Tuesday/Thursday-Friday	**Dilutant**	Semen 150µL	Semen 150µL	RPMI 1640 150µL	RPMI 1640 150µL
		**Virus or Mock**	500 ng p27 SIVsmB7	CEMx174 Supernatant	500 ng p27 SIVsmB7	CEMx174 Supernatant
**36-37****	Monday-Tuesday	**Dilutant**	Semen 150µL	Semen 150µL	RPMI 1640 150µL	RPMI 1640 150µL
		**Challenge Virus**	800 $TCID_{50}$ SIVmac251	800 $TCID_{50}$ SIVmac251	800 $TCID_{50}$ SIVmac251	800 $TCID_{50}$ SIVmac251
	Thursday-Friday	**Dilutant**	Semen 150µL	Semen 150µL	RPMI 1640 150µL	RPMI 1640 150µL
		**Virus or Mock**	500 ng p27 SIVsmB7	CEMx174 Supernatant	500 ng p27 SIVsmB7	CEMx174 Supernatant
**38 Rest Week**	Monday-Tuesday/Thursday-Friday	**Dilutant**	Semen 150µL	Semen 150µL	RPMI 1640 150µL	RPMI 1640 150µL
		**Virus or Mock**	500 ng p27 SIVsmB7	CEMx174 Supernatant	500 ng p27 SIVsmB7	CEMx174 Supernatant
**39-40**	Monday-Tuesday	**Dilutant**	Semen 150µL	Semen 150µL	RPMI 1640 150µL	RPMI 1640 150µL
		**Challenge Virus**	3200 $TCID_{50}$ SIVmac251	3200 $TCID_{50}$ SIVmac251	3200 $TCID_{50}$ SIVmac251	3200 $TCID_{50}$ SIVmac251
	Thursday-Friday	**Dilutant**	Semen 150µL	Semen 150µL	RPMI 1640 150µL	RPMI 1640 150µL
		**Virus or Mock**	500 ng p27 SIVsmB7	CEMx174 Supernatant	500 ng p27 SIVsmB7	CEMx174 Supernatant

Total treatment volumes were supplemented with additional RPMI 1640 to reach 30% volume of semen in a total inoculum volume of 500µL each inoculation

Biweekly treatment refers to treatments occurring Monday/Thursday or Tuesday/Friday

** Refers to the start of high-dose challenge and the end of the low-dose challenge analysis period

Supplementary Table 2: Menstrual cycle status in the week(s) prior to infection

Animal ID	Condition 1	Condition 2	Week Viral Load Detected						Week -1						Week -2					
			Study Week	Cycle Day	Bleeding	Cycle Phase	Estradiol	Progesterone	Study Week	Cycle Day	Bleeding	Cycle Phase	Estradiol	Progesterone	Study Week	Cycle Day	Bleeding	Cycle Phase	Estradiol	Progesterone
1I1	Semen	SIVsmB7	3	36	No	Luteal	54.5	0.252	2	29	No	Luteal	66	0.316	1	22	No	-	30	<0.200
9H5	Semen	SIVsmB7	5	6	No	Follicular	49.9	<0.200	4	25	No	Luteal	43.7	0.386	3	18	No	Luteal	99.5	4.7
9J4	Semen	SIVsmB7	8	7	No	Follicular	103	0.38	7	29	No	Luteal	83.3	1.52	6	22	No	Luteal	28.5	9.62
1J5	Semen	SIVsmB7	12	4	No	Follicular	104	<0.200	11	22	No	Luteal	71.9	2.06	10	15	No	Luteal	85.9	8
2I5	Semen	SIVsmB7	16	12	No	Luteal	40.1	3.51	15	5	No	Follicular	94.6	<0.200	14	21	No	Luteal	89.6	0.595
BH73	Semen	CEMsup	3	17	No	Luteal	42.9	4.34	2	10	Yes	Follicular	142	<0.200	1	3	Yes	Follicular	31	<0.200
9L3	Semen	CEMsup	7	13	No	Follicular	356	0.251	6	16	No	Luteal	34.7	6.63	5	26	No	Luteal	50.3	0.677
4J8	Semen	CEMsup	7	1	No	Follicular	20	0.267	6	6	No	Follicular	158	0.325	5	9	No	Follicular	125	0.203
8G9	No Semen	SIVsmB7	4	2	No	Follicular	20.5	<0.200	3	19	No	Luteal	49.5	1.47	2	12	No	Luteal	68.6	2.84
BJ39	No Semen	SIVsmB7	4	3	No	Follicular	23.7	0.259	3	26	No	Luteal	23.7	2.16	2	19	No	Follicular	66.1	<0.200
7H7	No Semen	SIVsmB7	6	19	No	Luteal	52	5.78	5	12	No	Luteal	25.4	0.615	4	3	No	Follicular	63	<0.200
7H1	No Semen	SIVsmB7	8	9	No	Follicular	83.3	<0.200	7	1	Yes	Follicular	29.6	<0.200	6	18	No	Luteal	36.2	4.02
9J9	No Semen	SIVsmB7	11	12	No	Luteal	33.3	r5.03	10	5	No	Follicular	174	<0.200	9	24	No	Luteal	59.8	0.28
7I1	No Semen	SIVsmB7	12	13	No	Luteal	43.2	4.36	11	6	No	Follicular	195	0.2	10	31	No	Luteal	69.9	0.435
1H3	No Semen	CEMsup	2	1	Yes	Follicular	50.6	3.28	1	81	No	Luteal	39.7	2.25	0	75	-	Follicular	86.4	<0.200
9J7	No Semen	CEMsup	3	12	No	Luteal	25.1	3.95	2	5	No	Follicular	158	<0.200	1	26	No	Luteal	78.7	0.248
BJ58	No Semen	CEMsup	5	11	No	Follicular	91.9	<0.200	4	4	Yes	Follicular	43.5	<0.200	3	21	No	Luteal	26.6	1.81
2H8	No Semen	CEMsup	6	6	No	Follicular	62.1	<0.200	5	30	No	Luteal	26.3	<0.200	4	23	No	Luteal	29.2	1.88
BJ36	No Semen	CEMsup	8	9	No	Follicular	33.6	0.231	7	1	Yes	Follicular	27.3	0.668	6	15	No	Luteal	32.9	1.47
7I3	No Semen	CEMsup	12	6	No	Follicular	85.9	<0.200	11	33	No	Luteal	60	0.439	10	26	No	Luteal	51.8	3.92
BH70	No Semen	CEMsup	14	3	No	Follicular	62	<0.200	13	19	No	Luteal	75.7	2.73	12	12	No	Luteal	55.9	5.2
1H5	Semen	SIVsmB7	19	14	No	Luteal	25.4	6.14	18	6	No	Follicular	92.8	<0.200	17	24	No	Luteal	72.9	0.684
5H8	Semen	SIVsmB7	20	20	No	Luteal	23.2	2.69	19	13	No	Follicular	52.4	<0.200	18	5	No	Follicular	65.6	<0.200
4J5	Semen	SIVsmB7	22	10	No	Luteal	65.1	0.349	21	3	No	Follicular	63.3	<0.200	20	22	No	Luteal	58.6	6.87
SBR123	Semen	CEMsup	17	1	Yes	Follicular	47.5	<0.200	16	19	No	Luteal	106	11.2	15	12	No	Luteal	65.4	0.694
BJ08	Semen	CEMsup	18	8	No	Follicular	67.2	<0.200	17	1	Yes	Follicular	34.7	<0.200	16	26	No	Luteal	43.4	3.53
2H1	Semen	CEMsup	19	6	No	Follicular	101	<0.200	18	26	No	Luteal	60.5	1.68	17	19	No	Luteal	68.2	12.2
2J1	Semen	CEMsup	19	22	No	Luteal	106	2.62	18	14	No	Luteal	43.1	5.11	17	7	No	Follicular	125	<0.200
2H0	Semen	CEMsup	20	4	No	Follicular	85	<0.200	19	22	No	Luteal	44.2	2.37	18	14	No	Luteal	36.7	6.3
9H0*	No Semen	SIVsmB7	19	14	No	Luteal	39.2	4.64	18	6	No	Follicular	81.9	<0.200	17	25	No	Luteal	56.9	0.634
BE23	No Semen	SIVsmB7	21	14	No	Luteal	37.5	2.43	20	8	No	Follicular	99.5	<0.200	19	1	No	Follicular	59	<0.200
0H2[t]	No Semen	CEMsup	Abnormal Cycle - Never Infected																	

* Removed from log-rank analysis due pre-existing SIV specific antibody response

[t] Animal never become infected

Horizontal Line within table indicates separation of infections during high-dose challenge period as opposed to the low-dose challenge period of the experiment phase of the study

Supplementary Table 3: Flow cytometry results of PBMC immune cell subsets

Variables	Vehicle	Semen	p-values
%CCR5+ of C. Mem. CD4 T-Cells	11.3	7.1	0.0298*
%CCR5+ of CD4 T-Cells	7.3	4.4	0.0366*
%CD69 of C. Mem. CD4 T-Cells	3.8	3.3	0.163
%CD8 T-Cells	41.4	37.9	0.169
%CD16 of Monocytes	15.0	16.6	0.181
%CD40+ of mDC	1.1	2.3	0.194
%CD69+ of CD4 T-Cells	3.7	3.1	0.243
%FoxP3+ CD25+ of CD4 T-Cells	2.4	1.9	0.300
%CD14+ Monocytes	4.1	3.6	0.318
%CD4 T-Cells	51.6	53.3	0.356
%CD8 NK Cells	3.8	4.7	0.366
%CD11c mDCs	1.6	2.2	0.418
%CD83+ of mDC	1.9	2.0	0.440
%C. Memory of CD8 T-Cells	28.2	27.2	0.486
HLA-DR MFI on pDC	1452.5	1386.1	0.510
HLA-DR MFI on Monocytes	4988.0	4485.5	0.534
%CD40+ of pDC	1.3	1.3	0.534
%Naive of CD4 T-Cells	28.0	38.8	0.638
%Naive of CD8 T-Cells	23.8	25.5	0.651
%HLA-DR+ of CD4 T-Cells	5.7	5.2	0.665
%C. Memory of CD4 T-Cells	63.3	58.5	0.692
%CD69+ of CD8 T-Cells	6.7	8.5	0.720
%CD16+ of NK Cells	66.7	66.9	0.749
%E. Memory of CD4 T-Cells	2.1	2.7	0.763
%E. Memory of CD8 T-Cells	42.5	43.9	0.777
%HLA-DR+ of CD8 T-Cells	7.4	6.4	0.792
%CD69+ of NK Cells	17.1	16.9	0.807
%CD83+ of pDC	1.4	1.3	0.807
%CD69/HLA-DR+ of CD8 T-Cells	0.6	0.6	0.821
%CD69/HLA-DR+ of CD4 T-Cells	0.4	0.4	0.836
HLA-DR MFI on mDC	1402.5	1319.9	0.895
%CD123 pDC	0.3	0.3	0.955

Supplementary Table 4: Spearman Correlations of Cervica Tissue Staining

Tissue	Variable	byVariable	Spearman ρ	Prob>|ρ|
Ectocervix	IFNe	CD123	0.8671	0.0003
Ectocervix	Ki67-LP	Ki67	0.8671	0.0003
Ectocervix	HLA-DR	CD4	0.8392	0.0006
Endocervix	CD68	FoxP3	0.7626	0.0022
Ectocervix	Ki67	Mx1	0.7552	0.0045
Ectocervix	Mx1-LP	Ki67	0.7413	0.0058
Ectocervix	Mx1-Epi	IFNe	0.7203	0.0082
Endocervix	CD68	IFNe	0.6835	0.0088
Endocervix	HLA-DR	IFNe	0.6659	0.0113
Ectocervix	Ki67-LP	Mx1-LP	0.6993	0.0114
Endocervix	IFNe	Mx1	0.6352	0.0171
Endocervix	FoxP3	IFNe	0.6264	0.0191
Ectocervix	Ki67-Epi	Mx1	0.6573	0.0202
Ectocervix	Mx1-LP	CD68	0.6503	0.0220
Endocervix	FoxP3	HLA-DR	0.6000	0.0261
Ectocervix	Mx1-LP	Mx1	0.6294	0.0283
Endocervix	CD4	FoxP3	0.6154	0.0284
Endocervix	CD68	HLA-DR	0.5824	0.0318
Ectocervix	Mx1-Epi	CD123	0.6154	0.0332
Endocervix	HLA-DR	Mx1	0.5560	0.0419
Endocervix	CD68	Mx1	0.5473	0.0458
Ectocervix	Ki67-LP	Mx1	0.5734	0.0513
Ectocervix	HLA-DR	CD68	0.5594	0.0586
Ectocervix	Mx1-Epi	Mx1	0.5594	0.0586
Ectocervix	Ki67-Epi	Ki67-LP	0.5524	0.0625
Ectocervix	IFNe	Mx1	0.5455	0.0666
Ectocervix	Mx1	CD123	0.5315	0.0754
Ectocervix	Ki67	CD123	0.5315	0.0754
Ectocervix	Mx1-LP	CD4	0.5035	0.0952
Ectocervix	Ki67-Epi	Mx1-LP	0.5035	0.0952

LP=Lamina Propria
Epi=Epithelium

Supplementary Table 5 : Antibodies used for flow cytometric staining

	Marker	Clone	Company
FoxP3 Regulatory T-Cells	FoxP3	236A/E7	Ebioscience
	CD25	BC96	Biolegend
	CD4	L200	BDBioscience
	CD3	SP34-2	BDBioscience
	**Marker**	**Clone**	**Company**
pDC/mDC Maturation	CD123	7G3	BDBioscience
	HLA-DR	G46-6	BDBioscience
	CD3	SP34-2	BDBioscience
	CD14	M5E2	Biolegend
	CD8	SK1	Biolegend
	CD20	2H7	Biolegend
	CD11c	S-HCL-3	BDBioscience
	CD83	HB15e	BDBioscience
	CD40	5C3	BDBioscience
	**Marker**	**Clone**	**Company**
NK/T-Cell Activation	CD69	FN50	BDBioscience
	HLA-DR	G46-6	BDBioscience
	CD95	DX2	BDBioscience
	CD28	CD28.2	BDBioscience
	CD25	M-A251	BDBioscience
	CD3	SP34-2	BDBioscience
	CD4	L200	BDBioscience
	CD25	BC96	Biolegend
	CD16	3G8	BDBioscience
	CD8	SK1	Biolegend
	CCR5	3A9	BDBioscience
