# Supplementary material for: Repeated semen exposure decreases cervicovaginal SIVmac251 infection in rhesus macaques
Source: Nat Commun. 2019 Aug 21;10:3753. doi: 10.1038/s41467-019-11814-5 (PMC6704120; doi:10.1038/s41467-019-11814-5)
Supplement: Supplementary file 3 — Reporting Summary [file 41467_2019_11814_MOESM3_ESM.pdf]

nature research

Corresponding author(s): Luis J. Montaner

Last updated by author(s): Aug 1, 2019

Reporting Summary

Nature Research wishes to improve the reproducibility of the work that we publish. This form provides structure for consistency and transparency in reporting. For further information on Nature Research policies, see Authors & Referees and the Editorial Policy Checklist.

Statistics

For all statistical analyses, confirm that the following items are present in the figure legend, table legend, main text, or Methods section.

n/a	Confirmed	
☐	☒	The exact sample size (*n*) for each experimental group/condition, given as a discrete number and unit of measurement
☐	☒	A statement on whether measurements were taken from distinct samples or whether the same sample was measured repeatedly
☐	☒	The statistical test(s) used AND whether they are one- or two-sided *Only common tests should be described solely by name; describe more complex techniques in the Methods section.*
☐	☒	A description of all covariates tested
☐	☒	A description of any assumptions or corrections, such as tests of normality and adjustment for multiple comparisons
☐	☒	A full description of the statistical parameters including central tendency (e.g. means) or other basic estimates (e.g. regression coefficient) AND variation (e.g. standard deviation) or associated estimates of uncertainty (e.g. confidence intervals)
☐	☒	For null hypothesis testing, the test statistic (e.g. *F*, *t*, *r*) with confidence intervals, effect sizes, degrees of freedom and *P* value noted *Give P values as exact values whenever suitable.*
☒	☐	For Bayesian analysis, information on the choice of priors and Markov chain Monte Carlo settings
☒	☐	For hierarchical and complex designs, identification of the appropriate level for tests and full reporting of outcomes
☐	☒	Estimates of effect sizes (e.g. Cohen's *d*, Pearson's *r*), indicating how they were calculated

Our web collection on statistics for biologists contains articles on many of the points above.

Software and code

Policy information about availability of computer code

Data collection	Microsoft Excel, v16.16 6
Data analysis	SAS, GraphPad Prism v6.03

For manuscripts utilizing custom algorithms or software that are central to the research but not yet described in published literature, software must be made available to editors/reviewers. We strongly encourage code deposition in a community repository (e.g. GitHub). See the Nature Research guidelines for submitting code & software for further information.

Data

Policy information about availability of data

All manuscripts must include a data availability statement. This statement should provide the following information, where applicable:

- Accession codes, unique identifiers, or web links for publicly available datasets
- A list of figures that have associated raw data
- A description of any restrictions on data availability

All figures in the manuscript are associated with raw data; the dataset summarized in figures is made available in Data Source File. Any additional data can be made available to members of the scientific community trough inter- institutional data use agreements. Requests should be addressed to the Corresponding Author.

Field-specific reporting

Please select the one below that is the best fit for your research. If you are not sure, read the appropriate sections before making your selection.

☒ Life sciences ☐ Behavioural & social sciences ☐ Ecological, evolutionary & environmental sciences

October 2018

Life sciences study design

All studies must disclose on these points even when the disclosure is negative.

Sample size	No formal sample size calculation was performed. The sample size (N= 32) was estimated based on a 2x2 factorial design (N=8/group), using a vaginally infectious dose titer (of challenge viral stock) with a known rate of infection in control animals. Specifically, the infectious dose used was based on prior in vivo titration of the infectious SIV251 stock used. The sample size used was adequate to detect a change in rate of infection based on viral stock experience of infectious dose and expected rate of infection in control animals when compared for effects of semen or SIVB7 particle exposure by 16 vs. 16 animals log-rank comparisons.
Data exclusions	One of 32 outcomes was excluded in primary analysis. The justification for this exclusion is described in the manuscript text: "To confirm that the infection outcomes analyzed were independent of potentially protective intravaginal antiviral responses, one animal in the SIVsmB7 treatment group (9H0), which developed SIV-specific IgA cross-reactive to both SIVmac and SIVsmm (Fig. S3B), was censored from final analysis. Another animal (BJ39) in the same grouping developed peripheral blood SIV-specific T-cell responses prior to infection (Fig. S3A) yet this animal was not censored due to a lack of a priori evidence that these responses are protective from vaginal challenge."
Replication	Multiple time points were assessed to ensure reproducibility. Following primary 2x2 factorial analysis in 32 animals indicating an effect by repeated semen exposure, we (1) compared control and semen alone groups to reconfirm that the observed effect was also present in the subgroup (included as Supplemental Figure), and (2) analyzed an independent group of animals pre-treated in identical fashion to identify factors that could support an antiviral effect as observed in primary analysis.
Randomization	Animals were randomized into all four primary groups. Consistent with randomization an equal distribution of MHC-I alleles was present between semen/no semen groups (see Table 1). Experimental manipulations were performed twice a week (conditioning, etc.) and animals were divided into two groups of 16 animals each (16 on Monday-Thursday/16 on Tuesday-Friday) with equal number of randomly assigned animals from each group.
Blinding	All treatments and sample analysis were blinded. All treatments were prepared by an independent laboratory (Dr. Sariol) and provided to the animal care team on the day of administration under a color coding (actual treatment was blinded to animal care team). All samples collected from animals and subsequently analyzed were blinded as to group assignment to corresponding collaborating laboratories (SIV viral loads, CD4 counts, hormone levels, immunocytochemistry on tissues, cytokine measures, anti-SIV titers, T-cell responses, flowcytometry, etc.).

Reporting for specific materials, systems and methods

We require information from authors about some types of materials, experimental systems and methods used in many studies. Here, indicate whether each material, system or method listed is relevant to your study. If you are not sure if a list item applies to your research, read the appropriate section before selecting a response.

Materials & experimental systems

n/a	Involved in the study	
☐	☒	Antibodies
☐	☒	Eukaryotic cell lines
☒	☐	Palaeontology
☐	☒	Animals and other organisms
☒	☐	Human research participants
☒	☐	Clinical data

Methods

n/a	Involved in the study	
☒	☐	ChIP-seq
☐	☒	Flow cytometry
☒	☐	MRI-based neuroimaging

Antibodies

Antibodies used	FoxP3 (236A/E7) Ebioscience, CD25 (BC96) Biolegend, CD4 (L200) BDBioscience, CD3 (SP34-2) BDBioscience, CD123 (7G3) BDBioscience, HLA-DR (G46-6) BDBioscience, CD14 (M5E2) Biolegend, CD3 (SP34-2) BDBioscience, CD8 (SK1) Biolegend, CD20 (2H7) Biolegend, CD11c (S-HCL-3) BDBioscience, CD83 (HB15e) BDBioscience, CD40 (5C3) BDBioscience, CD69 (FN50) BDBioscience, HLA-DR (G46-6) BDBioscience, CD95 (DX2) BDBioscience, CD28 (CD28.2) BDBioscience, CD25 (M-A251) BDBioscience, CD3 (SP34-2) BDBioscience, CD4 (L200) BDBioscience, CD25 (BC96) Biolegend, CD16 (3G8) BDBioscience, CD8 (SK1) Biolegend, CCR5 (3A9) BDBioscience,
Validation	NHP Reagent Resource provided validation for each antibody use.

Eukaryotic cell lines

Policy information about cell lines

Cell line source(s)	Cell lines provided by collaborator (E.K, C.S.) from the University of Puerto Rico

Authentication	None of the cell lines were authenticated
Mycoplasma contamination	Cell Lines were tested mycoplasma
Commonly misidentified lines (See ICLAC register)	*Name any commonly misidentified cell lines used in the study and provide a rationale for their use.*

Animals and other organisms

Policy information about studies involving animals; ARRIVE guidelines recommended for reporting animal research

Laboratory animals	Female Rhesus Macaques (Macaca mulatta) were used between ages 3 and 6 years old
Wild animals	The study did not involve wild animals.
Field-collected samples	The study did not involve samples collected from the field.
Ethics oversight	The animals were housed at the AAALAC-accredited facilities of the Animal Resources Center, UPR-MSC. All animal procedures were approved by the UPR-MSC, Institutional Animal Care and Use Committee (IACUC), and comply with the Guide for the Care and Use of Laboratory Animals. Animal Welfare Assurance Number: A3421, Protocol number: 3380113. Before experimental procedures, animals were single housed, as approved by the IACUC.

Note that full information on the approval of the study protocol must also be provided in the manuscript.

Flow Cytometry

Plots

Confirm that:

☒ The axis labels state the marker and fluorochrome used (e.g. CD4-FITC).

☒ The axis scales are clearly visible. Include numbers along axes only for bottom left plot of group (a 'group' is an analysis of identical markers).

☒ All plots are contour plots with outliers or pseudocolor plots.

☒ A numerical value for number of cells or percentage (with statistics) is provided.

Methodology

Sample preparation	Flow cytometry was done using peripheral blood mononuclear cells derived from rhesus macaque via phlebotomy
Instrument	LSR II (BD Biosciences)
Software	Flowjo Flow Cytometry Software (FlowJo, LLC)
Cell population abundance	N/A (All analyses were conducted on unsorted PBMC)
Gating strategy	After excluding duplets and cell aggregates based forward scatter-height and area, a gate was applied to include lymphocytes and monocytes based on forward and side scattering. Gating for T cell subsets was based on CD3 and CD4/8 expression, with thresholds based on isotype control stainings. Monocyte and dendritic call subsets were gated based on the expression of HLA-DR and CD14 (monocytes) or absence of HLA-DR and expression of CD123/CD11c (pDC and mDC, respectively); gates were positioned based on isotype control stainings. Expression thresholds for markers of activation and differentiation in these cellular subsets were determined based on a combination of FMO and isotype staining.

☒ Tick this box to confirm that a figure exemplifying the gating strategy is provided in the Supplementary Information.
